# Metabolic response of intestinal microbiota to guar gum consumption

**DOI:** 10.3389/fnut.2023.1160694

**Published:** 2023-06-30

**Authors:** Claudia Barber, Carlos Sabater, Francisco Guarner, Abelardo Margolles, Fernando Azpiroz

**Affiliations:** ^1^Digestive System Research Unit, University Hospital Vall d’Hebron, Barcelona, Spain; ^2^Departament de Medicina, Universitat Autònoma de Barcelona, Bellaterra, Spain; ^3^Centro de Investigación Biomédica en Red de Enfermedades Hepáticas y Digestivas (Ciberehd), Madrid, Spain; ^4^Department of Microbiology and Biochemistry, IPLA-CSIC, Asturias, Spain; ^5^Health Research Institute of Asturias, ISPA, Asturias, Spain

**Keywords:** guar gum, food additive, prebiotic, microbiota, metagenomics

## Abstract

**Background:**

Guar gum is used extensively as a thickening agent in food, but it remains uncertain whether and to what extent it is fermented by colonic microbiota and whether it has microbiota modulatory properties.

**Aim:**

To determine the metabolic response of intestinal microbiota to guar gum consumption, specifically, the extent of initial fermentation and subsequent adaptation.

**Methods:**

Single-center, single arm, open label, proof-of-concept study testing the effect of guar gum on microbiota metabolism and adaptation. Healthy male subjects (*n* = 12) were administered gum guar (8 g/day) for 18 days. Outcomes were measured before, at initial and late administration: (a) anal gas evacuations (number/day); (b) digestive sensations (daily scales); and (c) fecal gut microbiota taxonomy and metabolic functions by shotgun sequencing.

**Results:**

At initial consumption, guar gum induced a transient increase in anal gas evacuations and digestive sensations; gas evacuation completely reverted upon continuous administration, whereas sensations reverted only in part. Guar gum induced moderate changes in human microbiota composition at both taxonomic and functional levels. Positive associations between effects on microbiota (proliferation of *Agathobaculum butyriciproducens* and *Lachnospira pectinoschiza*) and hedonic sensations were detected.

**Conclusion:**

Guar gum is metabolized by intestinal microbiota, and, upon continuous consumption, induces a selective adaptation of microbial taxonomy and function. These data highlight the potential interest of guar gum for novel prebiotic ingredient formulation.

## 1. Introduction

Guar gum is a natural indigestible, soluble polysaccharide of the galactomannan family, extracted from the seeds of the *Cyamopsis tetragonalobus* plant. Because of its potential to provide a highly viscous solution at relatively low concentrations (less than 1% w/v), it is used extensively as a thickening agent or stabilizer in food, particularly in dairy and bakery; guar gum is commonly found in ice cream, yogurt, salad dressing, gluten-free baked goods, sauces, kefir, and breakfast cereals. Related with its rheological properties, guar gum modulates small bowel absorption of nutrients, with beneficial effects on cholesterol metabolism and glycemic control (1–3). However, despite its extensive use, it remains uncertain whether and to what extent natural guar gum is fermented by colonic microbiota. Hence, our aim was to determine the metabolic reaction of intestinal microbiota in response to natural guar gum consumption, specifically, the extent of initial fermentation and subsequent adaptation. To address our aim, we applied state-of-the-art methodology, combing on-line measurements of intestinal gas production, as an index of microbiota metabolic activity, with fecal microbiota composition and functionality.

The issue addressed by our study may have important implications, because some indigestible food components, defined as prebiotics, are selectively utilized by host microorganisms and confer a health benefit (4). The relevance of the potential prebiotic effects of food additives has been highlighted by the European Food Safety Agency (EFSA) (5, 6). Indeed, some prebiotics, including a partially hydrolyzed guar gum derivative with low viscosity, exert beneficial effects in patients with functional digestive disorders, such as the irritable bowel syndrome (IBS) (7–12). These patients complain of digestive symptoms in the absence of detectable cause by conventional diagnostic tests; functional digestive disorders constitute about half of the consultations in the gastroenterology clinic and impose a heavy socioeconomic burden (13).

## 2. Materials and methods

### 2.1. Study design

**Figure 1:**
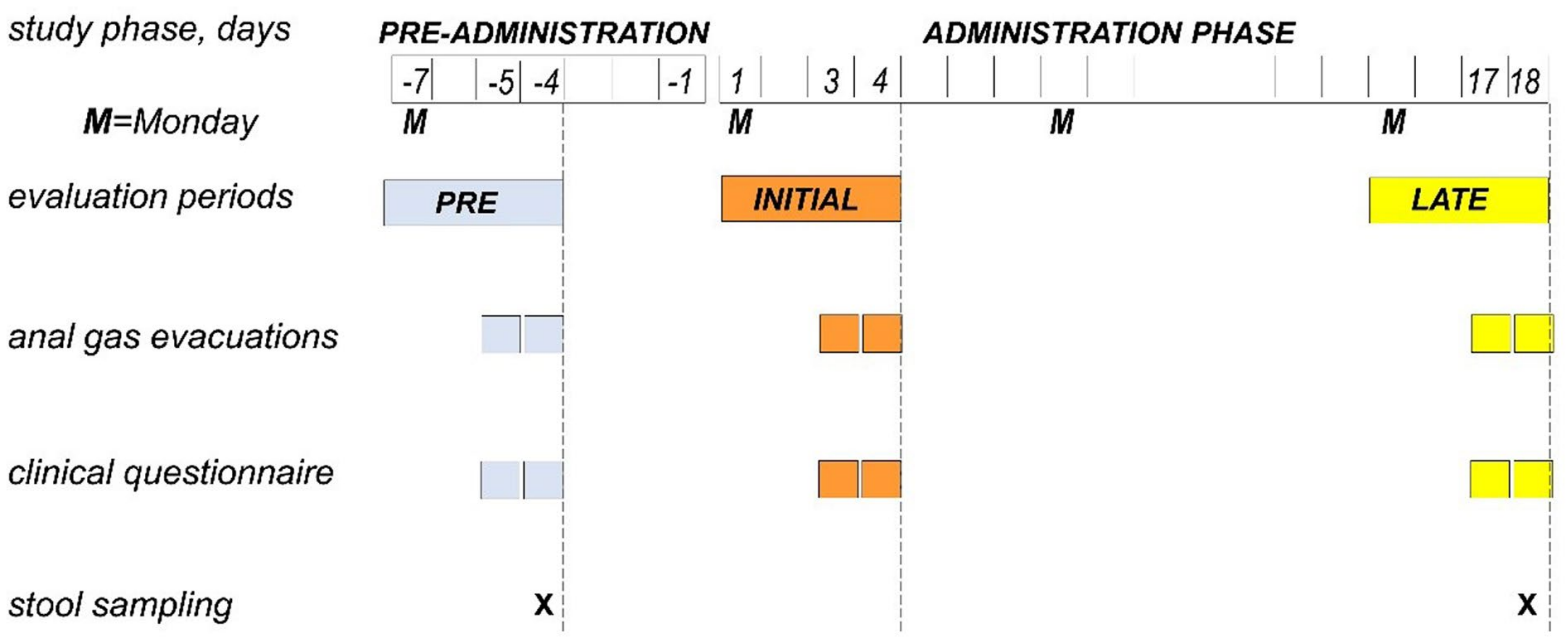
Experimental design and procedures. Single-center, single-arm, open-label, proof-of-concept study testing the effect of guar gum in healthy subjects. The study consisted of a pre-administration phase (7 days) and an administration phase of guar gum (8 g/day × 18 days). During 4-day evaluation periods (Monday to Thursday) in the pre-administration, initial administration and late administration, the diet was standardized, and the study outcomes were measured.

A single-center, single-arm, open-label, proof-of-concept study, testing the effect of guar gum on microbiota metabolism and adaptation, was performed in healthy men. The study consisted of pre-administration and 18-day administration phases (Figure 1). The following outcomes were measured during evaluation periods pre-administration, at initial administration and late administration: (a) anal gas evacuations (number/day); (b) subjective (digestive sensations) and objective consumer measures (bowel function) by daily scales; and (c) fecal gut microbiota taxonomy and metabolic functions by shotgun sequencing (Figure 1). The research was conducted according to the Declaration of Helsinki. The protocol for the study was previously approved by the Institutional Review Board of the University Hospital Vall d’Hebron, [Comitè d’Ètica d’Investigació Clinica, Vall d’Hebron Institut de Recerca; protocol number PR(AG)33/2021B approved February 1, 2021] and all participants provided written informed consent. The protocol was registered with ClinicalTrials.gov (NCT05195255).

### 2.2. Aims and sample size calculation

The primary aim of the study was to determine the change in gas production (evaluated by daily number of gas evacuations per anus using an event marker) from early versus late administration of guar gum. In a previous study, the number of daily anal gas evacuations during administration of a prebiotic (resistant dextrin) decreased from 21.1 evacuations at initial intake to 13.3 evacuation by 18 days administration (mean difference 7.80 evacuations per day, SD of difference 8.44) (14). Based on these data, it was estimated that a sample size of 12 subjects (paired) would be required to detect a change in the daily number of anal gas evacuations with 80% power and a 5% significance threshold. Participants were recruited following the inclusion and non-inclusion criteria described below (see the section 2.3). The secondary aims were to determine the effect of guar gum administration on the perception of digestive sensations (flatulence, bloating, abdominal distension, borborygmi, and abdominal discomfort/pain), digestive well-being and mood, bowel habit (stool weight, frequency, and consistency), and gut microbiota composition and functionality.

### 2.3. Participants

Healthy men without gastrointestinal symptoms or a history of gastrointestinal disorders participated in the study. Given the gender differences in the responses to meal ingestion (15, 16); in this proof-of-concept study only men were included for the sake of homogeneity. All participants were instructed to complete a clinical questionnaire based on Rome IV criteria, and only subjects not fulfilling criteria of functional gastrointestinal disorders (no symptom ≥ 2 on a 0–10 scale and normal bowel habits) were included. This questionnaire has been previously shown to discriminate patients from healthy subjects (17, 18). Non-inclusion criteria were: (i) antecedents of digestive surgery, excluding appendicectomy; (ii) antibiotic, prebiotic, or probiotic consumption during the previous 2 months; (iii) current use of any medications with potential central nervous system effects (e.g., antidepressants, anxiolytics, and opiate pain medications) or drugs that might modify gastrointestinal function; and (iv) change of dietary habits within the preceding 4 weeks.

### 2.4. Intervention

Guar gum powder (8 g/day; Now Foods; Bloomingdale, IL) was administered in the morning and evening (4 g per dose dissolved in water, juice or milk) during the 18-day administration phase.

### 2.5. Diet

During 4-day evaluation periods (Monday to Thursday) in the pre-administration phase (days-7 to -4), initial administration (days 1–4), and late administration phase (days 15–18), participants were put on a standard diet (Figure 1) restricted to the following foodstuffs: (i) meat, fish, fowl, and eggs; (ii) salad; (iii) rice, pasta, and bread; (iv) dairy products; and (v) strained orange juice, tangerine, pears, and apples. This low-residue diet (7 g fiber per day) was complemented with one portion per day of the following: whole crackers, lentils, chickpeas, beans, peas, artichoke, Brussels’ sprouts, banana, peach, or prunes; the portion size of each specific foodstuff was adjusted to contain 12 g fiber (18). The rest of the study participants consumed their habitual diet (Figure 1). During the study, participants were asked to avoid fermented dairy products (yogurts with living strains or probiotics-containing products), and any tablets, pills, or foods supplements containing fiber, pre or probiotics other than those provided. Dietary instructions were reinforced at each visit to ensure adherence to the study.

### 2.6. Outcomes

The following outcomes were measured during the last 2 days of the evaluation periods pre-administration (day-5 and -4), initial administration (days 3, 4), and late administration (days 17, 18; Figure 1).

#### 2.6.1. Number of anal gas evacuations

The number of anal gas evacuations during daytime was measured using an event marker (Hand Tally Counter No 101, Digi Sport Instruments, Shangqiu, China). Participants were instructed to carry the event marker during the day and register each passage of anal gas. This method has been previously used with reproducible and consistent results (17, 18).

#### 2.6.2. Clinical questionnaire

The participants were instructed to complete questionnaires at the end of each day (Figure 1), including the subjective and objective consumer measures, as follows. Subjective measures: (i) digestive sensations (subjective sensations of flatulence defined as anal gas evacuation, abdominal bloating defined as pressure/fullness, visible abdominal distension defined as girth increment, borborygmi defined as rumbling, and abdominal discomfort/pain) using analog scales graded from 0 (no sensation) to 10 (very severe sensation); (ii) hedonic sensations (digestive well-being and mood) using 10-point scale graded from +5 (extremely positive) to −5 (extremely negative). Objective measures: (iii) the number of bowel movements; (iv) stool form using the Bristol scale; and (v) stool weight using a balance provided by the investigator (digital weighing scale BT-32013, El Corte Ingles, Madrid, Spain). Participants received instructions to measure stool weight and to fill out scales by the end of the day. This questionnaire has been previously used and was shown to be sensitive to detecting the effects of dietary interventions in different populations (17, 18).

#### 2.6.3. Microbiota analysis

On the fourth day of the pre-adminstration period (day-4) and late administration period (day 18), fecal samples were collected, homogenized, and immediately frozen by the participants in their home freezers at −20°C (Figure 1). Total DNA extracted was submitted to an external sequencing service.^1^ Paired-end sequence reads (2 × 150 bp) showing an average minimum of 25 million reads per sample were generated using the Illumina NovaSeq system under accreditation according to the scope of BaseClear B.V. Reads were demultiplexed to generate FASTQ read sequence files using bcl2fastq2 (v2.18). The initial quality assessment was based on data passing the Illumina Chastity filtering. Subsequently, reads containing PhiX control signal were removed using an in-house filtering protocol. In addition, reads containing (partial) adapters were clipped (up to a minimum read length of 50 bp). The second quality assessment was based on the remaining reads using the FASTQC quality control tool version (v0.11.9).

Contaminant reads and low-quality sequences were separated *in silico* from microbial reads using Kneaddata (v0.7.4) and Trimmomatic (v0.39) software. With this aim, the minimum length of output reads was computed as 50% of the length of the input reads, considering a sliding window of 4:20. Bowtie2 (v2.4.2) was used to map metagenomic reads against the reference databases “*Homo sapiens* hg37 and human contamination Bowtie2” (v0.1), to remove host contamination.

Microbial taxonomic and functional profiling was performed using MetaPhlAn 3.0 (v3.0.4) and HUMAnN 3.0 (v3.0.0) pipelines (19, 20). ChocoPhlAn (version “mpa_v30_ChocoPhlAn_201901”) database containing clade-specific marker genes and UniRef90 (version “uniref90_201901”) protein database were used to perform taxonomic and functional analyses. The abundances of gene families and metabolic pathways were re-normalized and expressed in units of copies per million.

To characterize microbial diversity within a sample and between individuals, alpha and beta diversity estimators were computed. In this sense, beta-diversity analysis of microbial communities was performed following the Bray–Curtis dissimilarity method (21) implemented in Phyloseq R package (21). To filter rare taxa, only those clades showing at least 0.1% abundance in 50% of samples were considered. In addition, genes showing at least 1 million reads in at least 50% of samples were selected for the functional analysis. To illustrate major differences in microbiota composition, Principal Coordinate Analysis (PCoA) plots were generated using Microbiome R package (22).

MaAsLin2 method (23) was selected for the statistical analysis of taxonomic clades, microbial gene families, and metabolic pathways abundances. In this sense, statistical differences at pre-administration and late administration times were determined. Only those clades and genes showing *p_adj_* values (corrected by the Benjamini-Hochberg method) lower than 0.25 were considered to select only relevant differences. All statistical tests and models were performed on R (v4.1.1).

The raw sequences data were deposited in the Sequence Read Archive (SRA) of the NCBI (https://www.ncbi.nlm.nih.gov/sra, accessed on 3 February 2023) under BioProject code PRJNA906167.

### 2.7. Statistical analysis

Descriptive statistics were performed for the parameters measured. The means (±SE) of the variables measured were calculated. The Kolmogorov–Smirnov test was used to check the normality of the data distribution. Parametric normally-distributed data were compared by Student’s *t*-test for paired or unpaired data. Otherwise, the Wilcoxon signed-rank test was used for paired data, and the Mann–Whitney *U* test for unpaired data. Correlations between microbial composition and clinical metadata were expressed as Pearson correlation coefficients. In this sense, correlations between microbial clades modulated by guar gum administration and clinical parameters were determined. Tests were performed using a significance level of 5% (two-sided). For clarity, only significant differences are denoted in the figures.

## 3. Results

### 3.1. Demographics and study flow

Twelve healthy men (20–40 years age range; 20–29 kg/m^2^ body mass index range) were included in the study. Body weight ranged from 60 to 91 Kg, and hence, the absolute 8 mg daily dose of guar gum administered, corresponded to an actual dose ranging between 133 and 88 mg per Kg body weight per day. All the participants completed the studies, delivered the clinical questionnaires and fecal samples, and were included for analysis.

### 3.2. Number of anal gas evacuations

**Figure 2:**
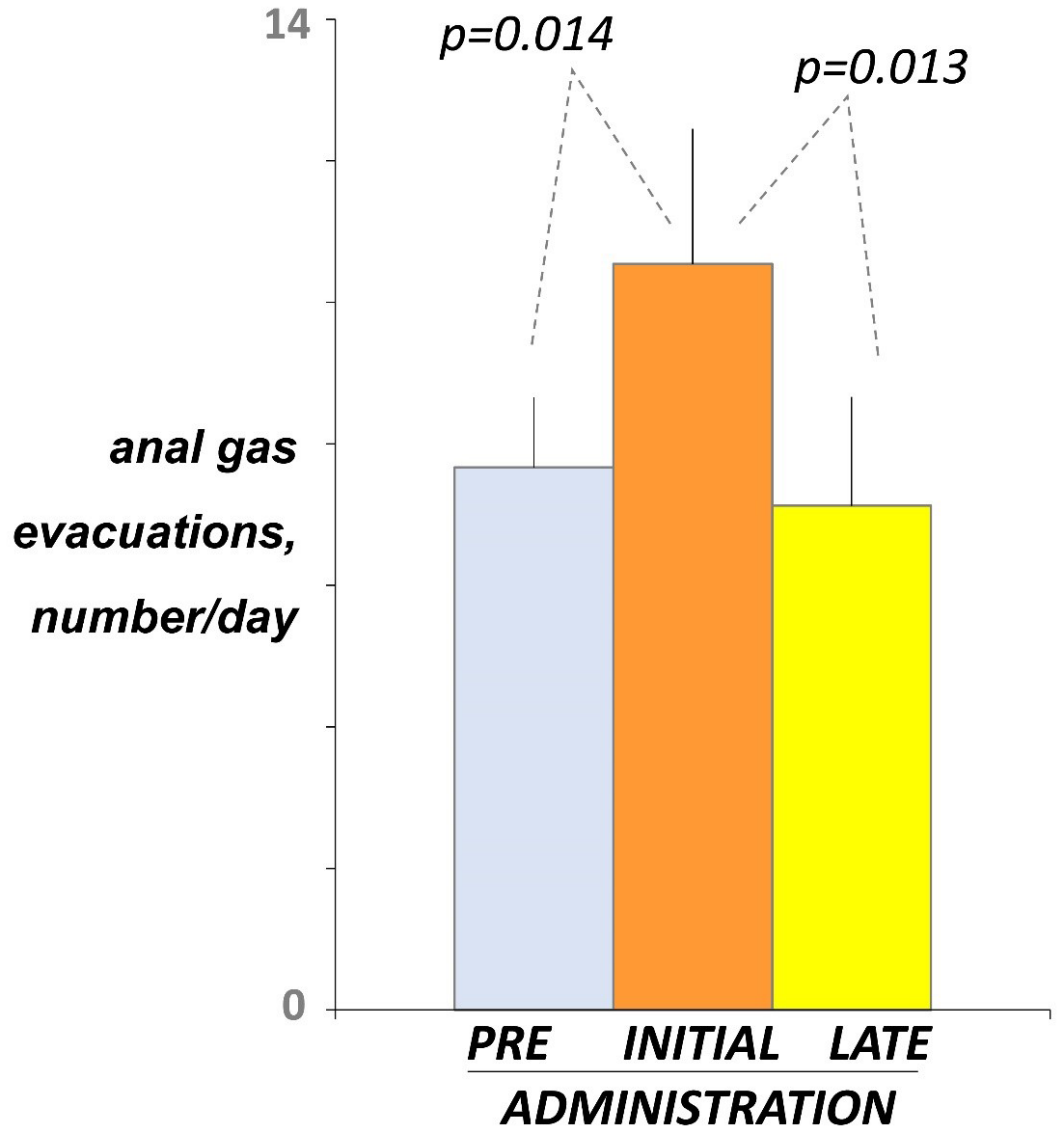
Intestinal gas evacuation. Number of daytime anal gas evacuations were measured by event marker. Figure shows values averaged over 2 consecutive days during pre-administration (days-5, -4), initial administration (days 3, 4), and late administration (days 17, 18). Note initial increase indicating guar gum fermentation, and later decline indicating microbiota adaptation.

Before administration (day-5 and -4 of the pre-administration phase), participants registered 7.7 ± 1.0 anal gas evacuations per day using the event marker (Figure 2). At initial exposure (days 3 and 4 of the administration phase), consumption of guar gum was associated with a small but significant increase in the number of anal gas evacuations (10.5 ± 1.9 evacuations/day; *p* = 0.014 vs. pre-administration; Figure 2). After continous administration (days 17 and 18), anal gas evacuation decreased and reverted to the level before administration (7.1 ± 1.5 evacuations/d; *p* = 0.013 vs. early administration; Figure 2). No differences were observed between the two consecutive evaluation days during each evaluation period.

### 3.3. Digestive sensations

**Figure 3:**
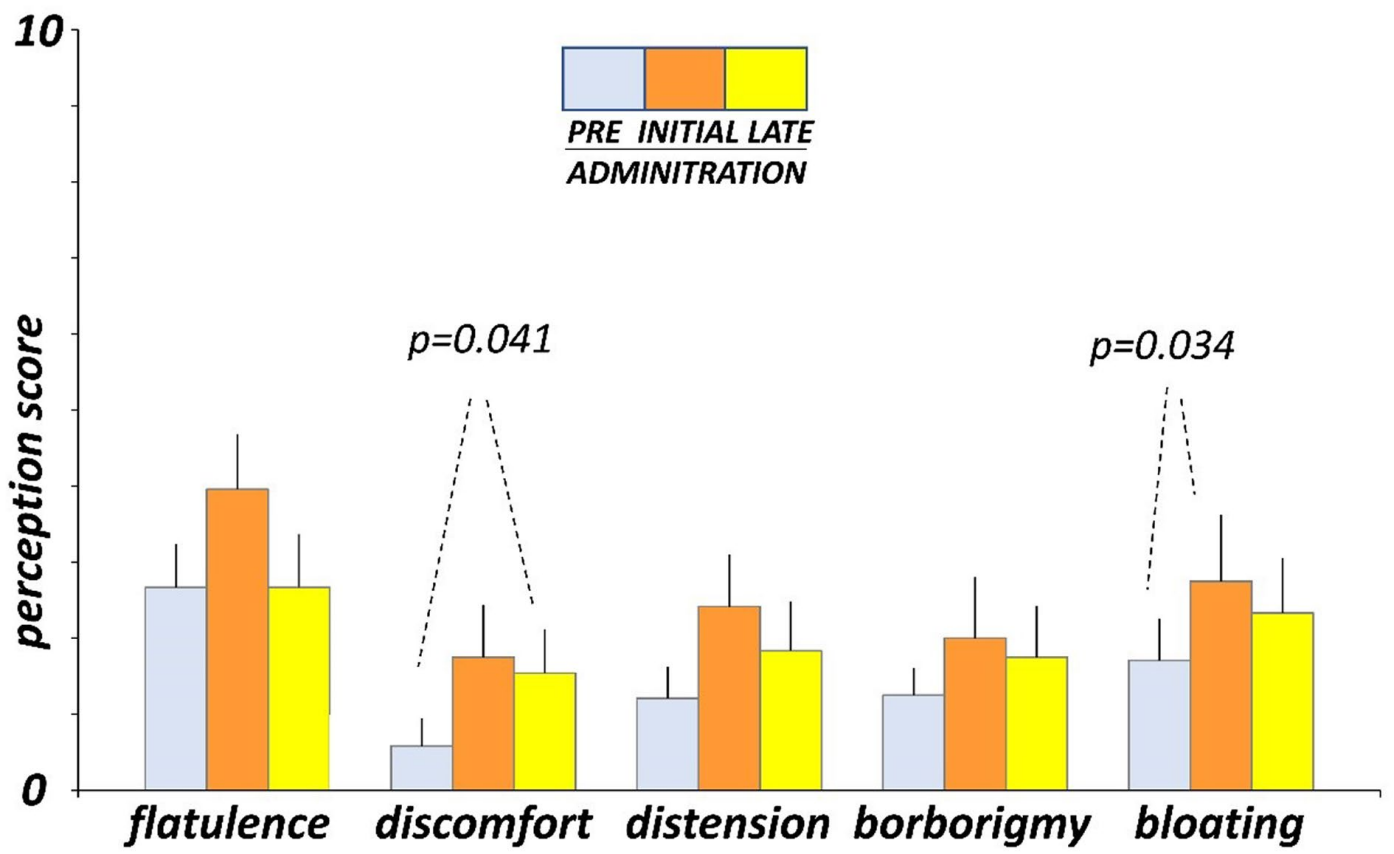
Digestive sensations. Sensations were measured by 0 (none) to 10 (very severe) scales. Figure shows values averaged over two consecutive days during pre-administration (days-5, -4), initial administration (days 3, 4), and late administration (days 17, 18).

Before administration (day-4 and -5), participants marked on the daily scales low scores of digestive sensations (Figure 3). At initial exposure (days 3 and 4), consumption of guar gum was associated with a mild increase in digestive sensation, which was statistically significant for abdominal bloating (*p* = 0.034 vs. pre-administration; Figure 3). After continuous administration (days 17 and 18), digestive sensations tended to decrease, but the differences between initial and late administration were not statistically significant (Figure 3). Interestingly, flatulence sensation paralleled intestinal gas evacuation and reverted to the level before administration, but the other sensations remained above pre-ingestion level; the differences between late ingestion and pre- ingestion levels were small and did not reach statistical significance, except for abdominal discomfort (*p* = 0.041). No significant differences were detected between the two consecutive evaluation days during each evaluation period.

### 3.4. Hedonic sensations

**Figure 4:**
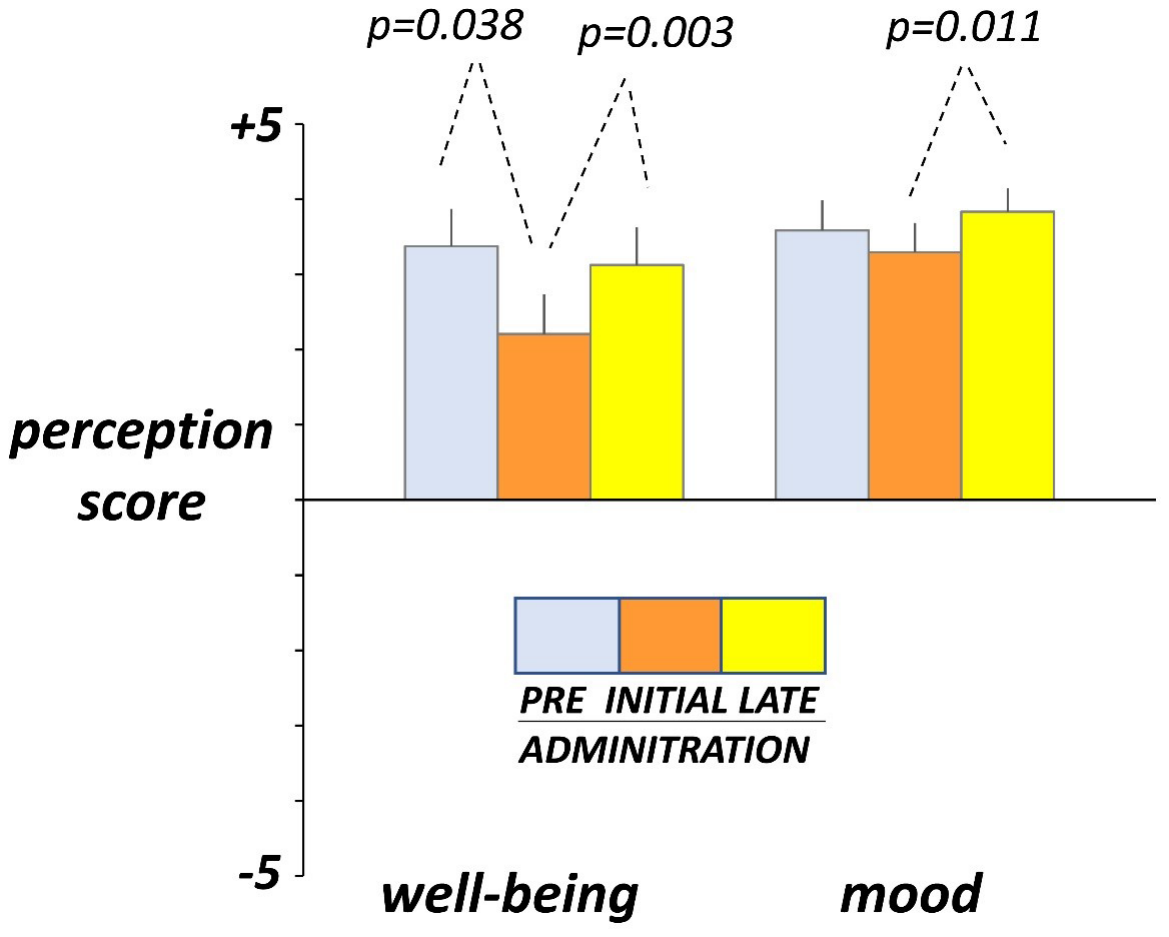
Hedonic sensations. Sensations were measured by +5 (very positive) to −5 (very negative) scales. Figure shows values averaged over two consecutive days during pre-administration (days -5, -4), initial administration (days 3, 4), and late administration (days 17, 18). Note initial drop and later recovery.

Hedonic sensations moved opposite to digestive sensations. Before administration (day-4 and -5), participants reported on the daily scales sensation of digestive well-being and positive mood (Figure 4). At initial exposure (days 3 and 4), consumption of guar gum was associated with a small but significant drop in digestive well-being *p* = 0.038 vs. pre-administration) and light, non-significant impairment of mood (Figure 4). By the late administration phase (days 17 and 18), both digestive well-being and mood significantly increased (*p* = 0.003 and *p* = 0.011 vs. initial ingestion phase, respectively) and returned back to the pre-ingestion levels (Figure 4). No significant differences were detected between the 2 consecutive evaluation days during each evaluation period.

### 3.5. Bowel habit

**Figure 5:**
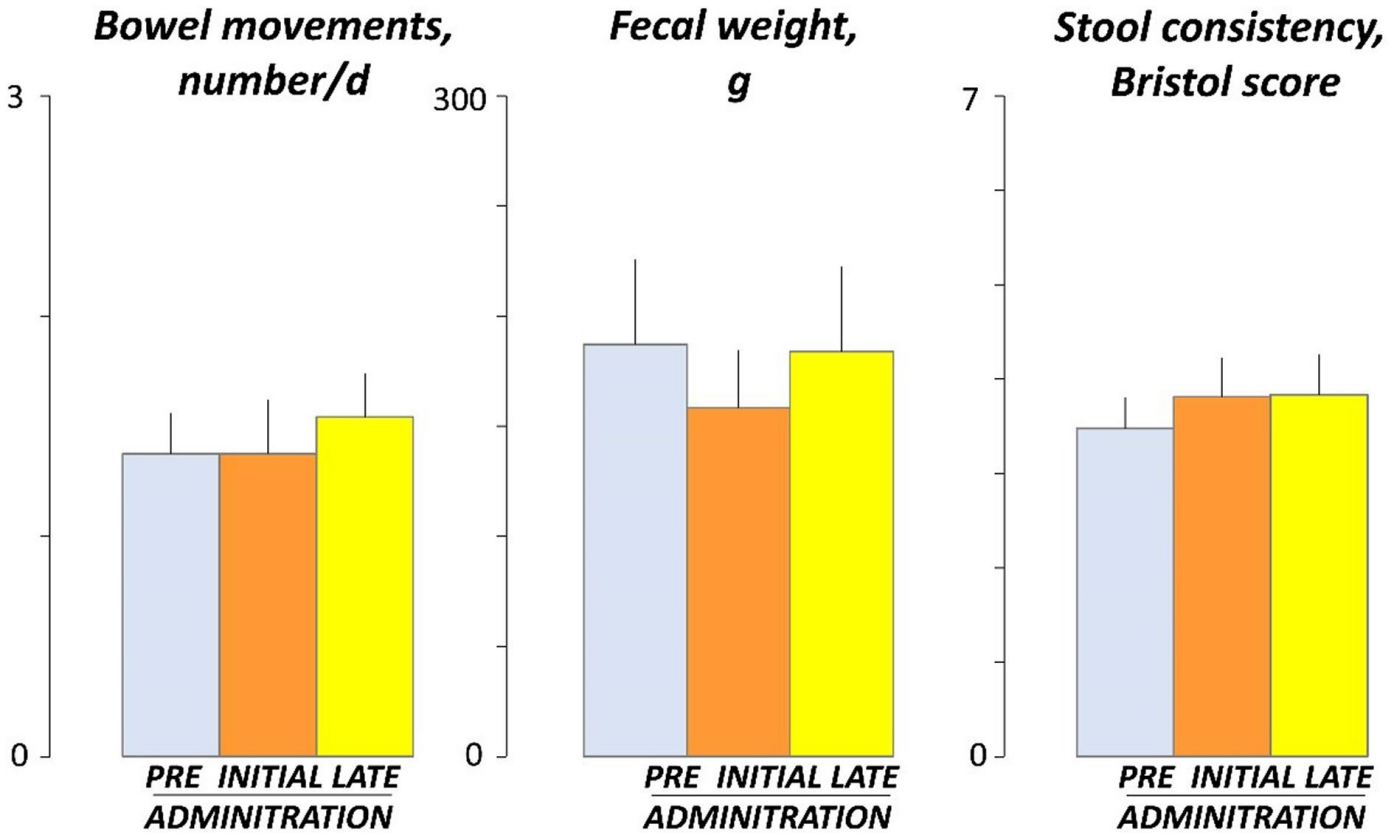
Bowel habit. Figure shows values averaged over 2 consecutive days during pre-administration (days -5, -4), initial administration (days 3, 4), and late administration (days 17, 18). There were no significant differences among pre, initial and late administration phases.

No effects of guar gum on normal bowel habit were observed: the number of stools per day, stool weight, and stool consistency, evaluated by the Bristol stool form scale, were similar before (days -4 and -5), at the beginning (days 3 and 4), and by the end (days 17 and 18) of the administration period (Figure 5).

### 3.6. Metagenomic analysis

**Figure 6:**
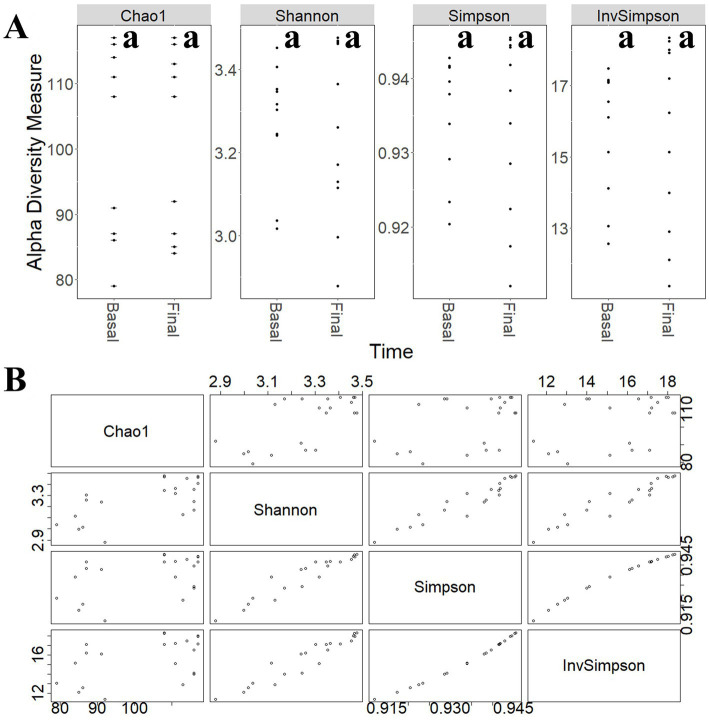
**(A)** Comparison of different alpha-diversity indicators (Chao 1, Shannon, Simpson, and Inverse Simpson) of the relative abundance of taxa determined at different intervention periods: pre-administration (basal) and late administration (final). **(B)** Relationship between different alpha-diversity estimators: Chao1, Shannon, Simpson, and Inverse Simpson indices. Most of these coefficients reflected similar patterns in the core microbiota. ^a^No statistically significant (*p* > 0.05) differences were found.

**Figure 7:**
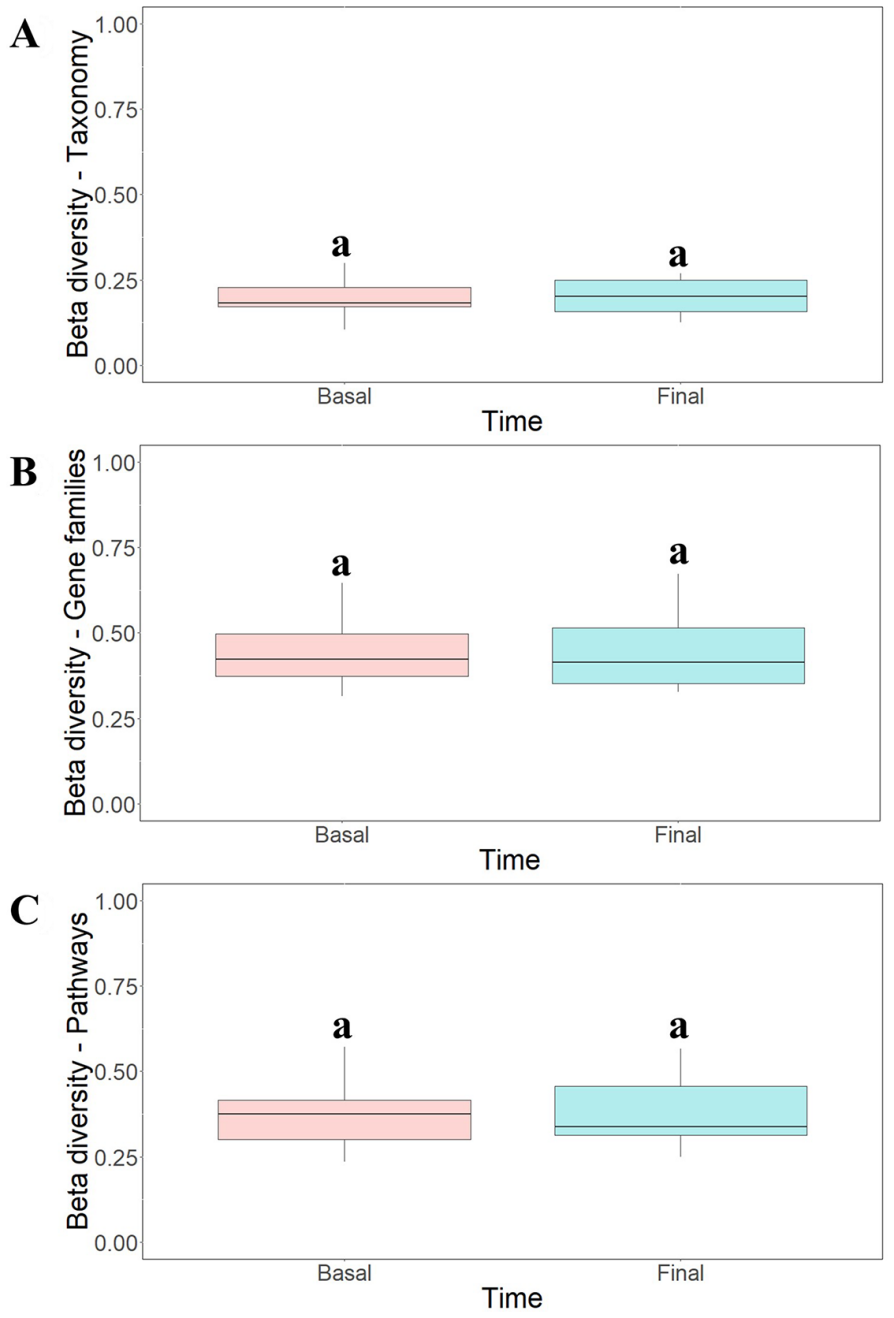
Beta-diversity analysis of taxonomic profiles **(A)**, gene families **(B)** and metabolic pathways **(C)** found in the microbiota of participants at different intervention periods: pre-administration (basal) and late administration (final). Bray-Curtis method was selected for the calculation. ^a^No statistically significant (*p* > 0.05) differences were found.

To study the modulatory effect of guar gum on human microbiota, a shotgun metagenomics analysis of fecal samples of participants collected at pre-and late administration periods was carried out. A taxonomic profiling of the microbiota was first performed. To measure the variability of species within a sample, Chao1 indexes, indicating the number of species represented by only one individual in the sample, were calculated. The global Chao1 index was 103 ± 14 while specific Chao1 indexes for pre- (103 ± 15) and late (104 ± 14) periods showed no major differences. Other alpha diversity estimators were compared, including Shannon, Simpson, and Inverse Simpson indexes (Figure 6A). These coefficients showed similar patterns in the core microbiota, confirming the results from the different analyses (Figure 6B). In general, no statistically significant (*p >* 0.05) changes in microbial diversity between pre-and late intervention periods were observed due to the high intra-sample variability. To further characterize microbial diversity between samples, beta diversity estimators based on Bray-Curtis distances were computed (Figure 7). Similar to the alpha diversity study, no statistically significant (*p* > 0.05) differences in the beta-diversity coefficients of samples collected at pre-and late intervention periods were observed (Figure 7A). Microbiota samples were then clustered according to the diversity distances calculated by the Bray–Curtis dissimilarity method (Supplementary Figure S1A). Some samples corresponding to the same intervention period were grouped, although several metagenomes were grouped with samples from different periods corresponding to the same participant. These results reflect the high inter-individual variability in the gut microbiota composition.

**Figure 8:**
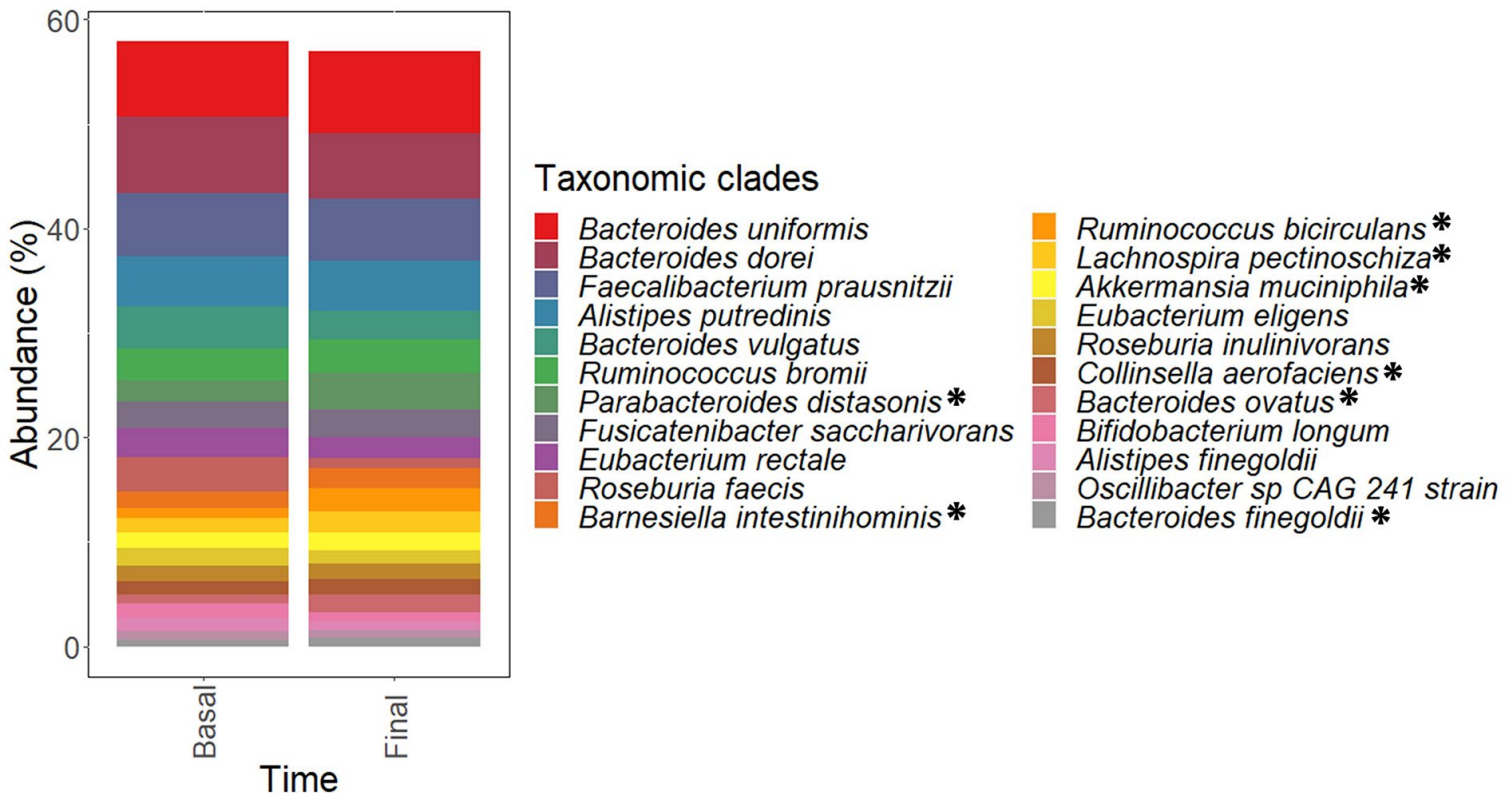
Most abundant species found in the microbiota of samples corresponding to pre-administration (basal) and late (final) administration periods. These species constitute the core microbiota of participants. Data are expressed as abundance percentages (%). ^*^Microbial species showing significantly (*p* < 0.05 and *p_adj_* < 0.25) higher abundances at late (final) administration period than pre-administration (basal) period.

The most abundant taxa found in fecal metagenomes included several Bacteroides species (*B uniformis, B. dorei*, and *B. vulgatus*) as well as *Faecalibacterium prausnitzii, Alistipes putredinis, Ruminococcus bromii*, and *Parabacteroides distasonis* (Figure 8). PCoA plots could not elucidate characteristic patterns in the complete microbiota profiles of samples according to the intervention time (pre-and late administration periods, Supplementary Figure S2A).

Functional analysis of metagenomes revealed no major differences in the gene count of samples collected at pre- (79,562 ± 21,175) and late (78,659 ± 22,213) administration periods. Similarly, no statistically significant (*p* > 0.05) differences in beta-diversity estimators of samples were observed (Figures 7B,C) and cluster analysis (Supplementary Figures S1B,C) and PCoA ordination plots (Supplementary Figures S2B,C) of functional data confirmed the high inter-individual variability observed in the taxonomic profiling.

Statistical analysis of taxonomic and functional data revealed significant differences in the abundances of some microbial clades (Tables 1–4) and genes (Table 5) after guar gum administration. Up to 35 microbial clades promoted by guar gum administration (Tables 1–4). Among these clades, *Parabacteroides*, *Ruminococcus*, *Barnesiella*, *Lachnospira*, *Akkermansia*, *Bacteroides*, *Collinsella* and *Agathobaculum* as well as *Oscillibacter* sp. 57 20 strain showed significantly (*p* < 0.05 and *p_adj_* < 0.25) higher abundances at final period than basal period. Therefore, these taxa were increased after the intervention. These clades showed abundance increments higher than 10% although most of these increments were moderate. Linear Discriminant Analysis (LDA) scores revealed differences in the abundances of these taxa among samples corresponding to pre-and late administration periods (Supplementary Figure S3). In contrast, other taxa including *Faecalibacterium*, *Alistipes*, *Fusicatenibacter*, and *Eubacterium* species showed no significant (*p* > 0.05) differences in their abundances.

**Table 1:** Microbial phyla modulated by guar gum administration.

*Microbial phyla showing higher abundances after late (final) intervention period*				
	Basal		Final	
Phylum	Mean	SD	Mean	SD
*Firmicutes* unclassified	0.57	0.71	**0.68**	0.89
*Verrucomicrobia*	1.52	3.02	**1.74**	3.42
*Proteobacteria*	0.91	0.87	**1.05**	0.88

Mean abundances and standard deviations (SD) of microbial phyla are compared at pre-administration (basal) and late (final) administration periods. These taxa showed significantly (*p* < 0.05 and *p_adj_* < 0.25) higher abundances at final period than basal period.

**Table 2:** Microbial families modulated by guar gum administration.

*Microbial families showing higher abundances after late (final) intervention period*				
	Basal		Final	
Family	Mean	SD	Mean	SD
*Prevotellaceae*	4.68	9.40	**8.37**	16.92
*Tannerellaceae*	3.17	2.12	**4.88**	2.91
*Acidaminococcaceae*	2.03	2.07	**2.93**	3.24
*Barnesiellaceae*	1.69	2.32	**2.24**	2.67
*Oscillospiraceae*	1.77	2.25	**2.03**	2.96
*Verrucomicrobiae*	1.52	3.02	**1.74**	3.42
*Akkermansiaceae*	1.52	3.02	**1.74**	3.42
*Coriobacteriaceae*	1.20	0.65	**1.49**	2.17

Mean abundances and standard deviations (SD) of microbial families are compared at pre-administration (basal) and late (final) administration periods. These taxa showed significantly (*p* < 0.05 and *p_adj_* < 0.25) higher abundances at final period than basal period.

**Table 3:** Microbial genera modulated by guar gum administration.

*Microbial genera showing higher abundances after late (final) intervention period*				
	Basal		Final	
Genus	Mean	SD	Mean	SD
*Ruminococcus*	4.76	5.64	**5.93**	6.56
*Parabacteroides*	3.17	2.12	**4.88**	2.91
*Phascolarctobacterium*	1.47	1.70	**2.18**	2.49
*Oscillibacter*	1.77	2.25	**2.03**	2.96
*Barnesiella*	1.55	2.26	**1.97**	2.63
*Lachnospira*	1.33	2.26	**1.95**	2.82
*Akkermansia*	1.52	3.02	**1.74**	3.42
*Collinsella*	1.19	0.65	**1.48**	2.17
*Agathobaculum*	0.42	0.42	**0.50**	0.53
*Coprobacter*	0.13	0.12	**0.27**	0.36
*Butyricimonas*	0.14	0.10	**0.15**	0.16

Mean abundances and standard deviations (SD) of microbial genera are compared at pre-administration (basal) and late (final) administration periods. These taxa showed significantly (*p* < 0.05 and *p_adj_* < 0.25) higher abundances at final period than basal period.

**Table 4:** Microbial species and strains modulated by guar gum administration.

*Microbial species showing higher abundances after late (final) intervention period*				
	Basal		Final	
Species/Strain	Mean	SD	Mean	SD
*Parabacteroides distasonis*	1.99	1.45	**3.48**	3.05
*Ruminococcus bicirculans*	1.03	1.41	**2.24**	5.63
*Barnesiella intestinihominis*	1.55	2.26	**1.97**	2.63
*Lachnospira pectinoschiza*	1.33	2.26	**1.95**	2.82
*Akkermansia muciniphila*	1.52	3.02	**1.74**	3.42
*Bacteroides ovatus*	0.86	0.96	**1.69**	2.23
*Collinsella aerofaciens*	1.19	0.65	**1.47**	2.16
*Bacteroides caccae*	1.03	1.25	**1.15**	1.37
*Parabacteroides merdae*	0.82	0.95	**0.92**	0.88
*Bacteroides finegoldii*	0.63	0.81	**0.78**	1.59
*Ruminococcus torques*	0.61	0.41	**0.71**	0.87
*Agathobaculum butyriciproducens*	0.42	0.42	**0.50**	0.53
*Oscillibacter sp.* 57 20 strain	0.92	1.26	**1.21**	1.17

Mean abundances and standard deviations (SD) of microbial species and strains are compared at pre-administration (basal) and late (final) administration periods. These taxa showed significantly (*p* < 0.05 and *p_adj_* < 0.25) higher abundances at final period than basal period.

**Table 5:** Number of microbial gene families and metabolic pathways showing the highest abundances at late intervention periods.

Microbial gene families modulated by guar gum administration		Microbial metabolic pathways modulated by guar gum administration	
Taxa	Frequency	Taxa	Frequency
*Parabacteroides distasonis*	3,602	*Bacteroides ovatus*	71
*Phascolarctobacterium faecium*	1773	*Parabacteroides distasonis*	71
*Bacteroides ovatus*	1,282	*Bacteroides caccae*	57
*Parabacteroides merdae*	1,113	*Collinsella aerofaciens*	44
*Oscillibacter sp.* 57 20	877	*Parabacteroides merdae*	44
*Ruminococcus torques*	507	*Phascolarctobacterium faecium*	38
*Bacteroides caccae*	505	*Ruminococcus bicirculans*	38
*Collinsella aerofaciens*	494	*Barnesiella intestinihominis*	35
*Ruminococcus bromii*	348	*Agathobaculum butyriciproducens*	20
*Butyricimonas virosa*	192	*Lachnospira pectinoschiza*	16
*Ruminococcus bicirculans*	185	*Oscillibacter sp.* 57 20	15
*Agathobaculum butyriciproducens*	103	*Bacteroides finegoldii*	8
*Barnesiella intestinihominis*	102	*Akkermansia muciniphila*	6
*Ruminococcus lactaris*	37	*Ruminococcus torques*	5
*Bacteroides finegoldii*	35	*Ruminococcus bromii*	2
*Lachnospira pectinoschiza*	35	*Butyricimonas synergistica*	1
*Oscillibacter sp.* CAG 241	24	*Butyricimonas virosa*	1
*Akkermansia muciniphila*	16	*Coprobacter fastidiosus*	1
*Coprobacter fastidiosus*	2	Total	473
Total	11,232		

Microbial metabolic pathways modulated by guar gum administration			
Function	Frequency	Function	Frequency
Unknown	16	L-arginine biosynthesis II	3
5-aminoimidazole ribonucleotide biosynthesis I	11	L-histidine degradation I	3
5-aminoimidazole ribonucleotide biosynthesis II	11	L-ornithine biosynthesis II	3
Superpathway of 5-aminoimidazole ribonucleotide biosynthesis	11	L-rhamnose degradation I	3
UMP biosynthesis I	11	Methylerythritol phosphate pathway II	3
L-valine biosynthesis	10	Pyridoxal 5-phosphate biosynthesis I	3
UMP biosynthesis II	10	Sucrose biosynthesis II	3
UMP biosynthesis III	10	Superpathway of adenosine nucleotides *de novo* biosynthesis I	3
Adenine and adenosine salvage III	9	Superpathway of adenosine nucleotides *de novo* biosynthesis II	3
Coenzyme A biosynthesis I	9	Superpathway of guanosine nucleotides *de novo* biosynthesis II	3
Guanosine ribonucleotides *de novo* biosynthesis	9	Superpathway of pyridoxal 5-phosphate biosynthesis and salvage	3
Queuosine biosynthesis I	9	Thiamine phosphate formation from pyrithiamine and oxythiamine	3
UDP-N-acetylmuramoyl-pentapeptide biosynthesis I	9	6-hydroxymethyl-dihydropterin diphosphate biosynthesis I	2
Coenzyme A biosynthesis II	8	Glycogen degradation II	2
Methylerythritol phosphate pathway I	8	Glycolysis III	2
Peptidoglycan biosynthesis I	8	Guanosine nucleotides degradation III	2
Superpathway of coenzyme A biosynthesis III	8	L-methionine biosynthesis III	2
Chorismate biosynthesis from 3-dehydroquinate	7	L-ornithine biosynthesis I	2
Chorismate biosynthesis I	7	NAD *de novo* biosynthesis I	2
dTDP-beta;-L-rhamnose biosynthesis	7	Pentose phosphate pathway I	2
Fatty acid biosynthesis initiation	7	Pentose phosphate pathway II	2
Inosine-5-phosphate biosynthesis II	7	Pyrimidine deoxyribonucleotide phosphorylation	2
L-histidine biosynthesis	7	Pyruvate fermentation to acetate and (S)-lactate I	2
L-isoleucine biosynthesis I	7	Pyruvate fermentation to acetate and lactate II	2
L-isoleucine biosynthesis III	7	Pyruvate fermentation to isobutanol	2
Peptidoglycan biosynthesis III	7	Seleno-amino acid biosynthesis	2
Phosphopantothenate biosynthesis I	7	Sucrose degradation III	2
Superpathway of branched chain amino acid biosynthesis	7	Superpathway of aromatic amino acid biosynthesis	2
UDP-N-acetylmuramoyl-pentapeptide biosynthesis II	7	Superpathway of pyrimidine nucleobases salvage	2
inosi$ne-5-phosphate biosynthesis I	6	4-amino-2-methyl-5-diphosphomethylpyrimidine biosynthesis II	1
L-lysine biosynthesis III	6	ADP-L-glycero-beta;-D-manno-heptose biosynthesis	1
L-methionine biosynthesis IV	6	biotin biosynthesis II	1
preQ0 biosynthesis	6	Calvin-Benson-Bassham cycle	1
Pyrimidine deoxyribonucleosides salvage	6	CMP-3-deoxy-D-manno-octulosonate biosynthesis	1
Superpathway of coenzyme A biosynthesis I	6	Entner-Doudoroff pathway I	1
Superpathway of guanosine nucleotides *de novo* biosynthesis I	6	GDP-mannose biosynthesis	1
UDP-N-acetylmuramoyl-pentapeptide biosynthesis III	6	L-arginine biosynthesis III	1
CDP-diacylglycerol biosynthesis I	5	L-arginine degradation XIII	1
CDP-diacylglycerol biosynthesis II	5	L-histidine degradation III	1
Folate transformations III	5	L-lysine biosynthesis II	1
Inosine 5-phosphate degradation	5	Lactose and galactose degradation I	1
L-lysine biosynthesis VI	5	Methanogenesis from acetate	1
Superpathway of L-serine and glycine biosynthesis I	5	Molybdopterin biosynthesis	1
tRNA charging	5	Myo-, chiro-and scyllo-inositol degradation	1
Adenosine deoxyribonucleotides *de novo* biosynthesis II	4	O-antigen building blocks biosynthesis	1
Cis-vaccenate biosynthesis	4	Pentose phosphate pathway	1
Folate transformations II	4	Purine ribonucleosides degradation	1
Glycogen biosynthesis I	4	S-adenosyl-L-methionine salvage I	1
Guanosine deoxyribonucleotides *de novo* biosynthesis II	4	Stachyose degradation	1
Peptidoglycan maturation	4	Sucrose degradation IV	1
UDP-N-acetyl-D-glucosamine biosynthesis I	4	Superpathway of L-aspartate and L-asparagine biosynthesis	1
Flavin biosynthesis I	3	Superpathway of L-threonine biosynthesis	1
Glycolysis IV	3	Superpathway of pyrimidine ribonucleotides *de novo* biosynthesis	1
Gondoate biosynthesis	3	Tetrapyrrole biosynthesis I	1
Isoprene biosynthesis I	3	Thiamine diphosphate salvage II	1
L-arginine biosynthesis I	3	**Total**	**473**

These gene families and metabolic pathways modulated by guar gum administration are summarized by bacterial species and metabolic function. These gene families and metabolic pathways showed significantly (*p* < 0.05 and *p_adj_* < 0.25) higher abundances at final period than basal period.

With regard to the functional analysis, the abundances of a total 11,232 microbial gene families and 473 microbial metabolic pathways significantly (*p* < 0.05 and *p_adj_* < 0.25) higher abundances at final period than basal period (Table 5). These genes and pathways corresponded to *Bacteroides*, *Parabacteroides*, *Phascolarctobacterium*, *Collinsella*, *Oscillibacter*, and *Ruminococcus* species in agreement with the statistical differences determined in the taxonomic analysis (Tables 1–4). These metabolic pathways involved a wide range of functions (Table 5), including amino acid and ribonucleotide biosynthesis and carbohydrate metabolism.

**Figure 9:**
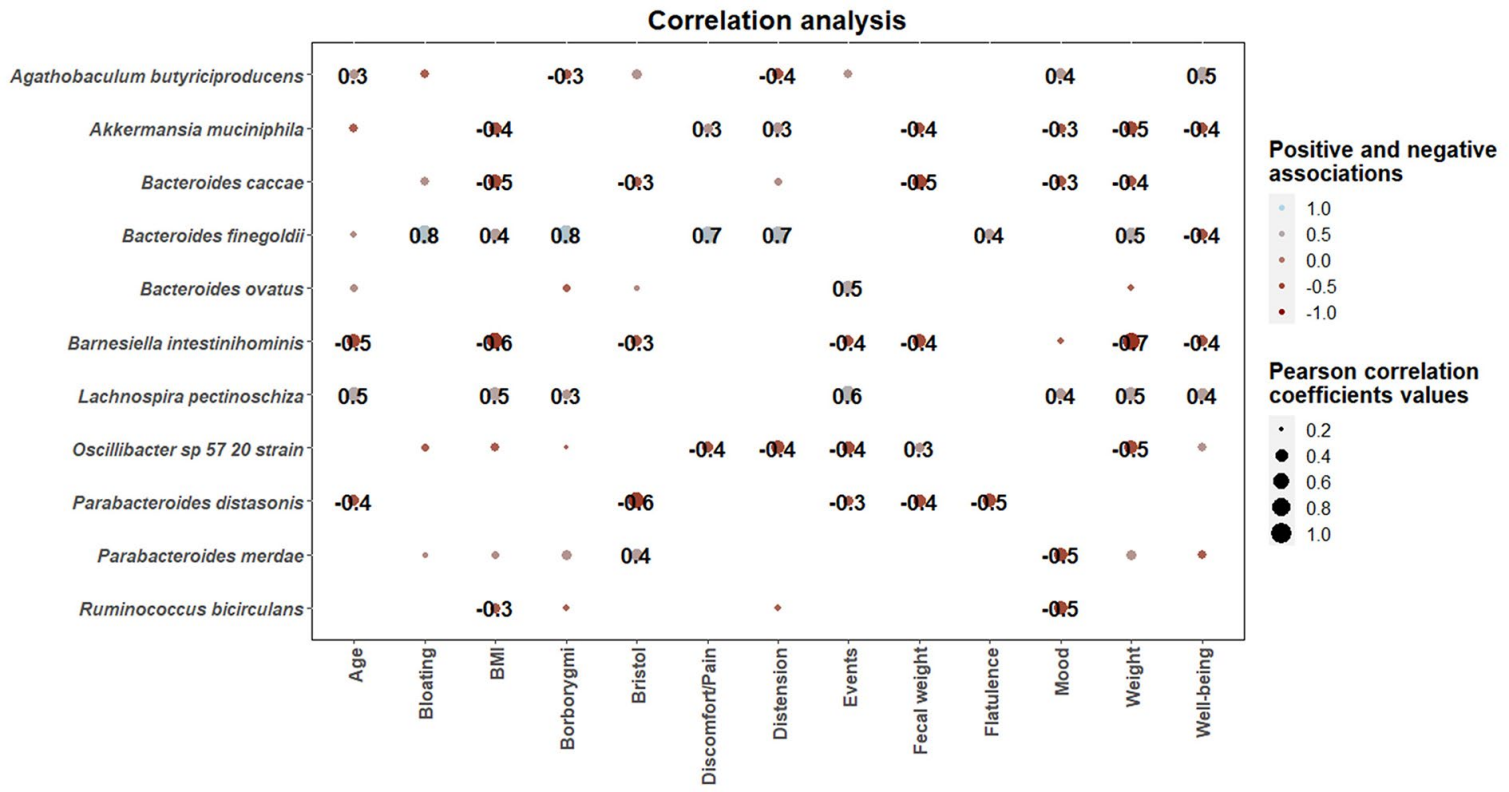
Correlation heatmaps showing the associations between microbial species modulated by guar gum administration and clinical parameters. Blue and red dots indicate positive and negative correlations expressed as Pearson correlation coefficients. Color intensity and dot size are in proportion to magnitude. These correlations were statistically significant (*p* < 0.05). BMI, body mass index.

Once statistical differences in microbial taxonomic and functional profiles at pre-and late administration periods were elucidated, statistically significant (*p* < 0.05) associations between microbial species modulated by guar guam and clinical metadata of participants were determined (Figure 9). In this regard, some microbial species including *Akkermansia muciniphila*, *Bacteroides caccae*, and *Barnesiella intestinihominis* showed negative association with BMI and fecal weight, and positive associations with abdominal discomfort/pain and distension. Similarly, *Bacteroides finegoldii* positive associations with borborygmi, abdominal discomfort/pain, distension, and flatulence. On the other hand, positive associations between *Agathobaculum butyriciproducens* and *Lachnospira pectinoschiza* and participant mood and well-being were found. *L. pectinoschiza* also showed positive associations with the number of anal gas evacuations.

## 4. Discussion

Our study indicates that natural guar gum serves as a substrate for intestinal microbiota, and continuous consumption induces a selective adaptation of metabolic functions, that may confer health benefits for the host.

Initial administration of guar gum was associated with an increase in intestinal gas production, reflecting its fermentation by intestinal microbiota. To note, the increase in gas production was full-blown by the third administration day, suggesting that guar gum is readily available for microbiota metabolism without pre-processing. Interestingly, the initial increase in gas production completely reverted upon continuous administration, reflecting a change in the fermentative pathways due to microbiota adaptation. The same phenomenon, initial fermentation and subsequent adaptation toward more efficient metabolic pathways, has been observed with other fibers with prebiotic properties (14, 24, 25). By contrast, poorly fermentable fibers, such as psyllium, do not induce detectable changes in intestinal gas evacuation, specifically the initial gas increase at initial administration (26).

Consumption of guar gum was initially associated with digestive sensations. In parallel to the decrease in gas evacuation upon continuous consumption, the sensation of flatulence reverted back to pre-administration level; however other sensations, particularly abdominal discomfort and bloating, still remained higher than before administration. These differences suggest that the origin of the sensations involves mechanisms related to gas, and other mechanisms possibly related to the bulk-forming effect of guar gum. Despite that guar gum did not influence fecal output, a potential effect on colonic biomass is plausible; indeed, other prebiotics produce a persistent increase in the volume of colonic biomass without changes in fecal output (14).

Guar gum administration induced changes at taxonomic and functional levels, and interestingly, these changes were observed only in specific microbial clades. Previous studies suggest that partially hydrolyzed guar gum derivatives have prebiotic properties (27). *In vitro* fermentation studies reported that partially hydrolyzed guar gum stimulates beneficial genera like *Bacteroides* and *Parabacteroides* (28), identified by 16S rRNA gene sequencing. In addition, 16S rRNA gene sequencing of microbiota samples from healthy volunteers revealed that partially hydrolyzed guar gum has been associated with an increase in the abundances of *Bacteroides*, *Faecalibacterium*, and *Ruminococcus* (8). These data are concordant with the effects of natural guar gum found in our study, which further show that these changes were also induced at functional level, including microbial gene families and metabolic pathways, and that correlate with clinical outcomes.

Some microbial clades promoted by guar gum administration, were also found to be promoted by fiber-based nutritional interventions in other studies. Specifically, *Parabacteroides* and *Ruminococcus* genera, and *L. pectinoschiza* species were promoted by a commercial preparation of resistant dextrin (14) while *Agathobaculum butyriciproducens* was promoted after fiber-enriched Mediterranean-type diet in healthy subjects, showing positive correlations with fecal weight and stool movements (29). Other dietary fibers such as psyllium husk show a different fermentative pattern compared to guar gum, leading to an increase in *Lactobacillus* and *Faecalibacterium* in chronically constipated women of reproductive age (29). However, psyllium administration resulted in an enrichment in *Ruminococcaceae*, similar to the one observed for *Ruminococcus* in the present work (30).

Overall, in our group of healthy subjects, the sensations were very mild, and guar gum was well tolerated. Nevertheless, given the adaptation induced on microbiota, it is plausible that natural guar gum may improve symptoms in patients with functional digestive disorders, as shown for other fibers with prebiotic properties, such as the partially hydrolized guar gum (7–12) and beta-galactooligosaccharide (31).

## 5. Limitations

This proof-of-concept study involved a small sample size, but still allowed to demonstrate the effect of guar gum on intestinal microbiota. Furthermore, the conclusions apply to healthy men, and it remains to be demonstrated whether they can be extended to women and to disease states, such as patients with functional digestive disorders. The intervention was relatively short, and we cannot ascertain whether longer administration may produce a more complete adaptation, improving bulk-related sensations and tolerance. Potential post-administration effects were not investigated, but given the adaptation induced on microbiota, it could be expected that some beneficial effects may persist for some time after administration.

## 6. Conclusion and inference

The present study shows that guar gum is well-tolerated, at least by healthy men, and exerts a selective modulatory effect on human microbiota at both taxonomic and functional levels. These data are highly relevant, considering the wide use of guar gum in food production, its metabolic benefits related to its modulatory effect on intestinal absorption, and its potential application for the treatment of functional digestive symptoms.

## Data availability statement

The data presented in the study are deposited in the NCBI - SRA repository (https://www.ncbi.nlm.nih.gov/), accession number PRJNA906167.

## Ethics statement

The studies involving human participants were reviewed and approved by Comitè d’Ètica d’Investigació Clinica, Vall d’Hebron Institut de Recerca; protocol number PR(AG)33/2021B approved February 1, 2021. The patients/participants provided their written informed consent to participate in this study.

## Author contributions

FA, FG, and AM: conceptualization. CB and CS: formal analysis, investigation, and methodology. FA and AM: project administration, resources, and validation. FA: supervision. FA and CS: writing original draft. AM and FG: writing—review and editing. All authors contributed to the article and approved the submitted version.

## Funding

This work was supported in part by the projects PID2021-122295OB-I00 (Ministerio de Ciencia e Innovación, Spain). Ciberehd is funded by the Instituto de Salud Carlos III.

## Conflict of interest

The authors declare that the research was conducted in the absence of any commercial or financial relationships that could be construed as a potential conflict of interest.

## Publisher’s note

All claims expressed in this article are solely those of the authors and do not necessarily represent those of their affiliated organizations, or those of the publisher, the editors and the reviewers. Any product that may be evaluated in this article, or claim that may be made by its manufacturer, is not guaranteed or endorsed by the publisher.

## Footnotes

^1^ www.BaseClear.com
