# Supplementary material for: Metabolic response of intestinal microbiota to guar gum consumption
Source: Front Nutr. 2023 Jun 30;10:1160694. doi: 10.3389/fnut.2023.1160694 (PMC10349393; doi:10.3389/fnut.2023.1160694)
Supplement: Supplementary file 1 [file Data_Sheet_1.docx]

Supplementary Material

Metabolic response of intestinal microbiota to guar gum consumption

Claudia Barber, M.D., Carlos Sabater, Ph.D.*, Francisco Guarner, M.D., Abelardo Margolles, Ph.D., Fernando Azpiroz M.D.

*** Correspondence:** Carlos Sabater: [carlos.sabater@csic.es](mailto:carlos.sabater@csic.es)

**
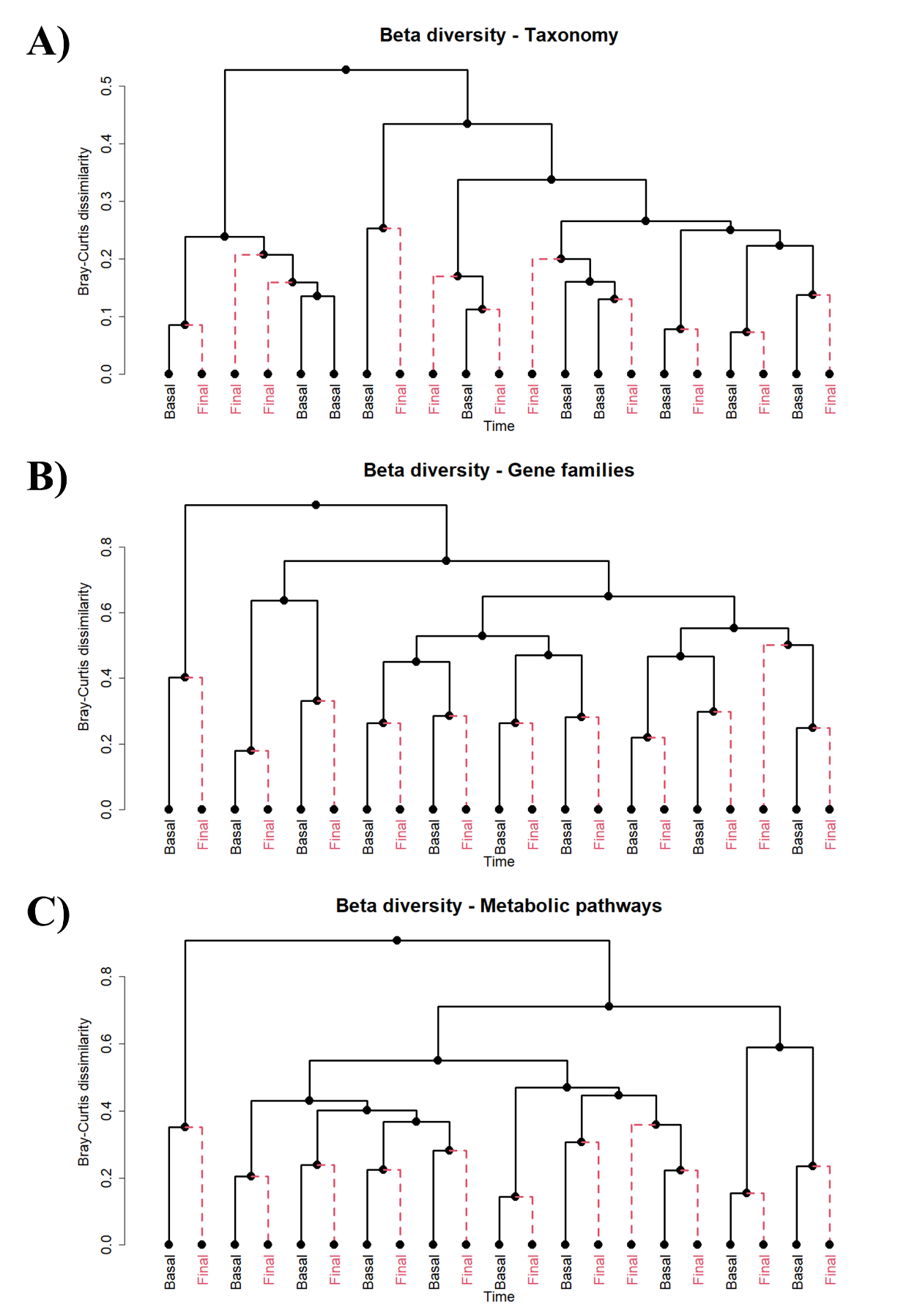
**

**Supplementary Figure S1.** Clustering of analysis of taxonomic profiles (**A**), gene families (**B**) and metabolic pathways (**C**) found in the microbiota of participants at different intervention periods: pre-administration (basal) and late administration (final). Bray-Curtis dissimilarity method was selected for the calculation. As it can be seen, few samples corresponding to the same intervention period were clustered together. The rest of samples were clustered together with metagenomes from the same participant taken at different periods, highlighting the role of interindividual variability.

**
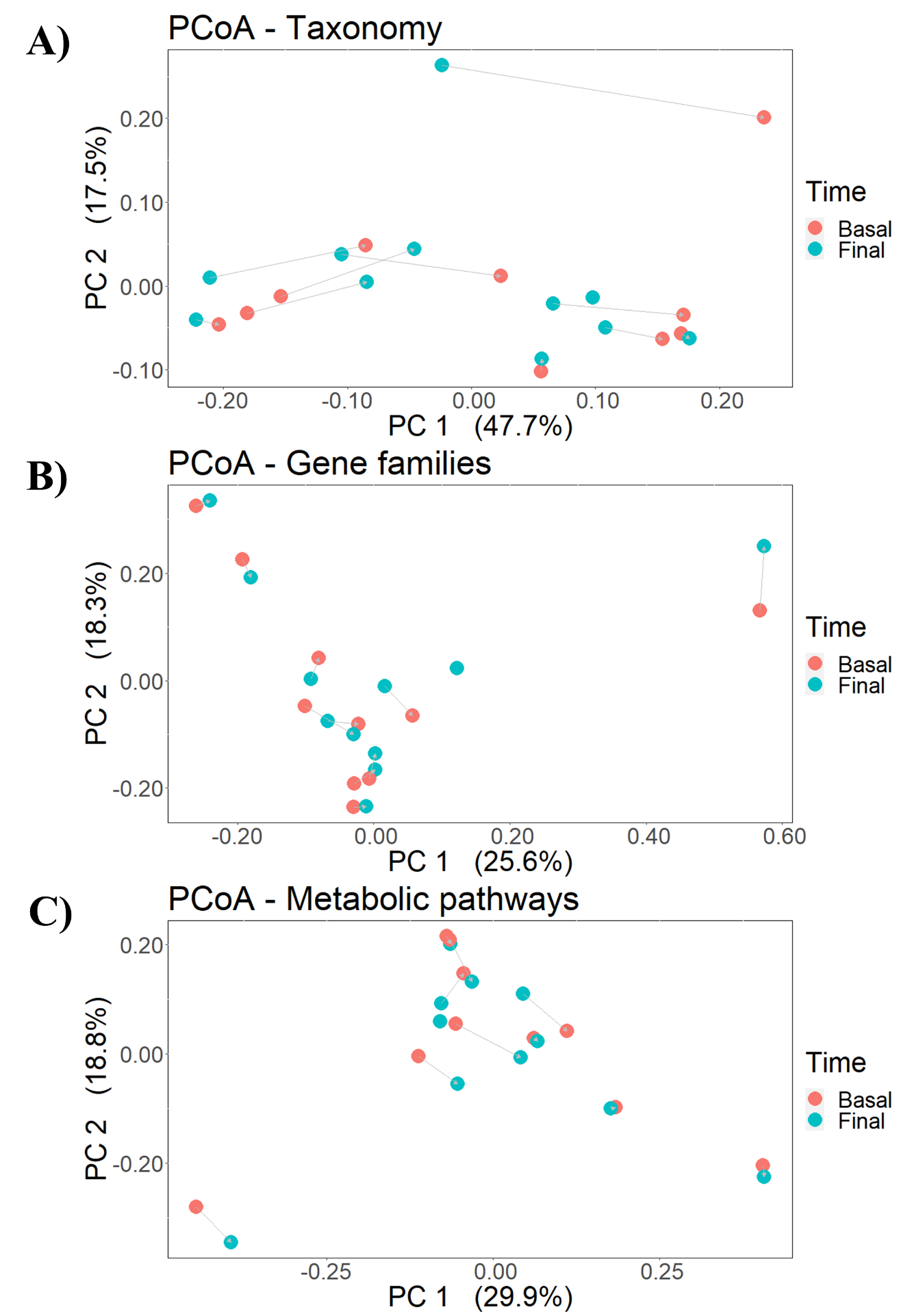
**

**Supplementary Figure S2.** Principal coordinates analysis (**PCoA**) of taxonomic profiles (**A**), gene families (**B**) and metabolic pathways (**C**) found in the microbiota of participants at different intervention periods: pre-administration (basal) and late administration (final). **PC:** principal coordinate. The percentage of variance explained by each PC is indicated in the axis.


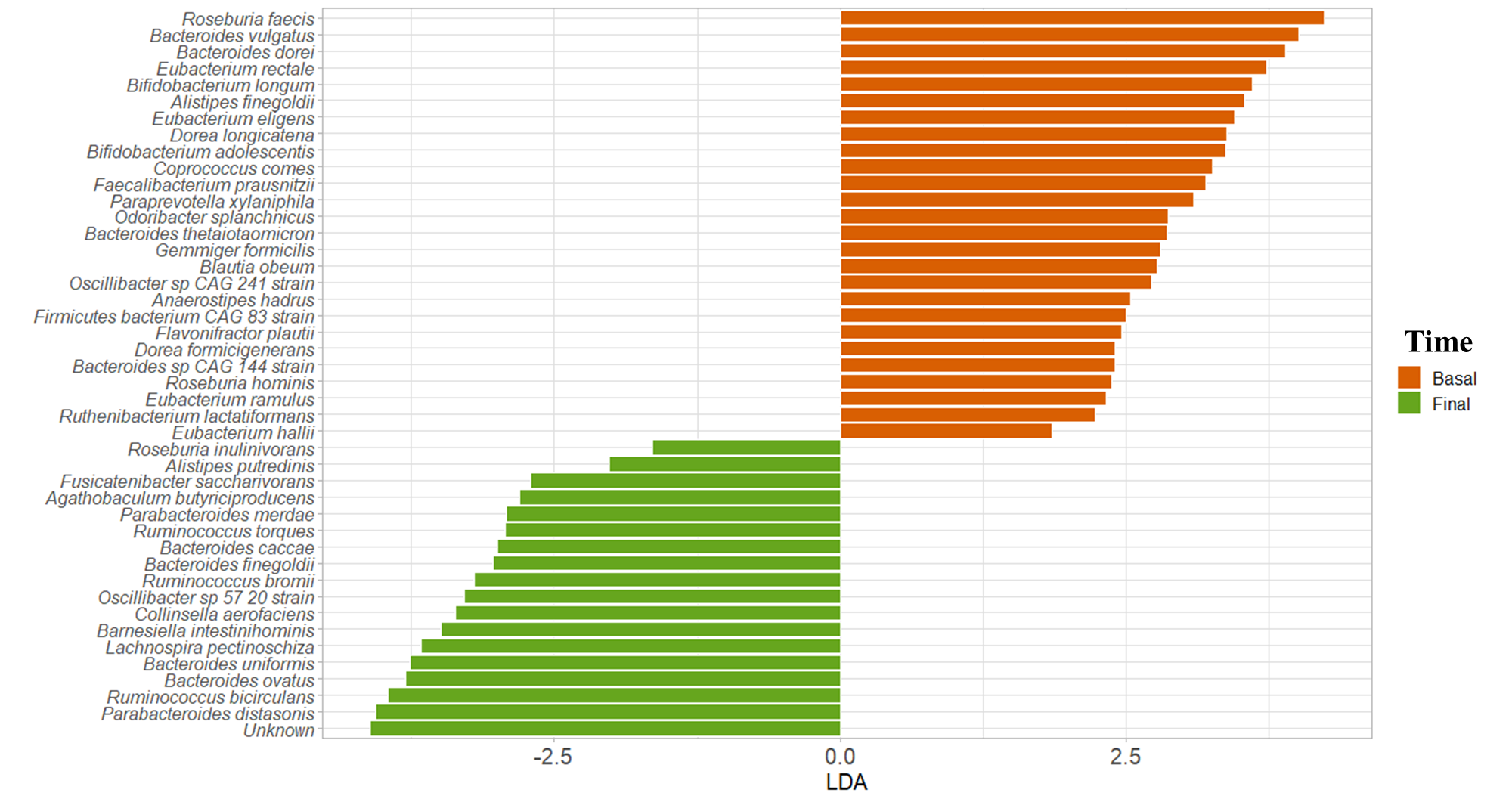


**Supplementary Figure S3.** Linear Discriminant Analysis (LDA) scores of differentially abundant microbial species and strains among samples corresponding to pre-administration (basal, red) and late (final, green) administration periods.
